# The MAP Kinase Phosphatase MKP-1 Modulates Neurogenesis via Effects on BNIP3 and Autophagy

**DOI:** 10.3390/biom11121871

**Published:** 2021-12-14

**Authors:** Yinghui Li, Marc W. Halterman

**Affiliations:** Department of Neurology, Renaissance School of Medicine, Stony Brook University, Stony Brook, NY 11794, USA; Yinghui.Li@stonybrookmedicine.edu

**Keywords:** MKP-1, neurogenesis, autophagy, BNIP3

## Abstract

Inherited and acquired defects in neurogenesis contribute to neurodevelopmental disorders, dysfunctional neural plasticity, and may underlie pathology in a range of neurodegenerative conditions. Mitogen-activated protein kinases (MAPKs) regulate the proliferation, survival, and differentiation of neural stem cells. While the balance between MAPKs and the family of MAPK dual-specificity phosphatases (DUSPs) regulates axon branching and synaptic plasticity, the specific role that DUSPs play in neurogenesis remains unexplored. In the current study, we asked whether the canonical DUSP, MAP Kinase Phosphatase-1 (MKP-1), influences neural stem cell differentiation and the extent to which DUSP-dependent autophagy is operational in this context. Under basal conditions, *Mkp-1* knockout mice generated fewer doublecortin (DCX) positive neurons within the dentate gyrus (DG) characterized by the accumulation of LC3 puncta. Analyses of wild-type neural stem cell (NSC) differentiation in vitro revealed increased *Mkp-1* mRNA expression during the initial 24-h period. Notably, *Mkp-1* KO NSC differentiation produced fewer Tuj1-positive neurons and was associated with increased expression of the BCL2/adenovirus E1B 19-kD protein-interacting protein 3 (BNIP3) and levels of autophagy. Conversely, Bnip3 knockdown in differentiated *Mkp-1* KO NSCs reduced levels of autophagy and increased neuronal yields. These results indicate that MKP-1 exerts a pro-neurogenic bias during a critical window in NSC differentiation by regulating BNIP3 and basal autophagy levels.

## 1. Introduction

Neurogenesis is a tightly controlled process occurring during embryonic development that persists in the adult brain, primarily within the subventricular zone (SVZ) and the hippocampal dentate gyrus (DG). Defects in neurogenesis have been linked to affective disorders and severe intellectual and behavioral deficits. During the initial phase of CNS development, the brain generates up to 250,000 neurons per minute [1]. Adult hippocampal neurogenesis in the DG is highly conserved across mammals [2]. Supported by the permissive environment of the neurovascular niche [3], neurogenesis generates approximately 700 new neurons per day, with a modest decline during aging [4]. Under conditions of mild physiological stress, cell-intrinsic responses and the release of brain-derived neurotrophic factor (BDNF) induces neurogenesis [5]. Conversely, genetic conditions, such as Down Syndrome, and age-related neurodegenerative conditions, such as Parkinson’s and Alzheimer’s diseases, are associated with a net reduction in neural stem cell (NSC) proliferation and neurogenesis [6,7,8]. Given the potential therapeutic benefit, enhancing induced neurogenesis under these and other pathological conditions remains an active area of investigation [8,9,10]. A more thorough understanding of the cell-intrinsic factors that modulate neurogenesis could inform new approaches to treat inherited and acquired disorders of the central nervous system (CNS).

Initially considered a key pathway for clearing long-lived proteins [11], autophagy also supports cell survival under nutrient starvation and other stress conditions by recycling energy substrates [12]. Autophagy involves the formation of the autophagosome, fusion with the lysosome, and the digestion of associated contents [13], with each step regulated by a hierarchy of autophagy-related genes. Recent evidence from studies of mice harboring mutations in autophagy-related genes implicates autophagy in the process of neurogenesis and neuronal differentiation via effects on β-catenin, Wnt, and Notch pathways [14]. In this context, autophagy supports cellular homeostasis via the elimination of protein aggregates and the degradation of senescent organelles, both crucial for NSC survival and differentiation during development and adulthood [15].

MAP kinase phosphatase-1 (MKP-1), an archetypal member of the MAPK dual-specificity phosphatase (DUSP) family, provides negative feedback on MAPK signaling with a bias towards inactivating the kinases p38 and JNK (c-Jun N-terminal kinase) [16]. In 2016, Wang et al. were the first to report a potential link between MKP-1 and autophagy, which indicates these effects are related to MAPK-dependent effects on the autophagy-related proteins ULK1 and BECN1 and the formation of the autophagosome-initiating complex [17]. MKP-1 regulates cell cycle progression and apoptosis by modulating mitochondrial function, oxidative stress, and autophagy [18]. MKP1 deficiency also promotes excessive mitophagy in models of ischemia-reperfusion (IR) injury [19]. MKP-1 plays a neuroprotective role in neurodegenerative diseases, including Alzheimer’s [20] and Huntington’s disease [21], both of which are characterized by neuron loss, impaired neurogenesis, and altered levels of autophagy [22,23]. MKP-1-dependent JNK inhibition improves the survival of cerebellar granule neurons and c17.2 mouse neural stem cells under conditions of endoplasmic reticulum stress [24]. Notably, MKP-1 expression is reduced in the SVZ and DG in a mouse model of Parkinson’s disease [25], while MKP-1 dysregulation is associated with the survival and proliferation of tumor progenitors within the glioblastoma stem cell niche [26].

In the current study, we investigated the tandem effects of MKP-1 on basal autophagy and neurogenesis. Our findings indicate that the loss of MKP-1 diminishes the neurogenic capacity of stem cells in the dentate gyrus and in differentiated neural stem cultures. In this context, we also demonstrate that MKP-1-dependent effects on neurogenesis are directly related to the expression of the BH3 protein BNIP3 and autophagic activity.

## 2. Materials and Methods

### 2.1. Animals

Mice were housed in specific pathogen-free (SPF) facilities and given ad libitum access to food and water in a 12 h light/dark cycle in a climate-controlled vivarium in an identical, non-enriched cage environment. *Mkp-1*+/− mice were provided by Dr. Bennett [27] under a materials transfer agreement with Bristol Meyer Squibb (Princeton, NJ, USA). Mice were bred to the C57BL6 background, and for these studies, male and timed pregnant female mice aged 12–14 weeks were used. Genotyping was performed using *Mkp-1* primers (fwd 5′-CTGACAGTGCAGAATCCGGA-3′, rev 5′-CTATGAAGTCAATAGCCTCGTTGA-3′) and neomycin resistance gene (neo^r^) primers (fwd 5′-ACTGTGTCGGTGGTGCTAATGAGA-3′, rev 5′-TACCGGTGGATGTGGAATGTGT-3′) (Supplementary Figure S1).

### 2.2. Immunohistochemistry (IHC)

Mice were perfused with saline, followed by 4% paraformaldehyde (PFA), and hemispheres were isolated and stored in PFA, 20% sucrose, and 30% sucrose for 24 h each. After 7 days, brains were frozen and sectioned at 25 microns with Leica SM2010R Sliding Microtome (Leica, Nussloch, Germany). Sections were blocked in blocking buffer with 10% goat serum at room temperature for 1 h and then incubated with primary antibody at 4 °C overnight, followed by incubation with corresponding Alexa Fluor secondary antibody (A-11075, A-11012, 1:1000–1:500, ThermoFisher Scientific, Waltham, MA, USA) for 1 h at room temperature. After rinsing with PBS, sections were mounted with ProLong Gold Antifade Mountant with DAPI (P36935, ThermoFisher Scientific) on slides and cover slipped. Primary antibodies used were doublecortin (DCX, 1:1000, AB2253, Millipore Sigma, Burlington, MA, USA) and LC3 (1:50, APG8B, Abgent, San Diego, CA, USA). Coronal sections from matched regions (Bregma −2.46~−1.46 mm, *n* = 3) were used from each sample to quantify DCX-positive cells and LC3 puncta numbers in the CNS. IHC stained sections were blinded, imaged using Lionheart FX automated microscope (BioTek, Winooski, VT, USA), and evaluated for the numbers of DCX positive cells and LC3 puncta numbers within DG using ImageJ. The sum of DCX counts on both sides of DG was used as the DCX positive cell number for each brain. LC3 puncta were counted in five 100 pixels by 100 pixels regions in each DG, and the sum of the numbers was used as the puncta number of a brain.

### 2.3. Neural Stem Cells

Neural stem cells (NSCs) were collected from embryonic day 14 (E14) WT and *Mkp-1* knockout mice following the protocol provided by Azari et al. [28]. In brief, females at day 14 of gestation were sacrificed, and embryos were separated from the uterine horns and maintained on ice-cold HBSS (ThermoFisher Scientific). Brains were removed from the skull, and hemispheres were separated. The cortical ganglionic eminences were removed, put into 1 mL NeuroCult NSC basal medium (#05700, STEMCELL Technologies, Cambridge, MA, USA), and dissociated by pipetting. Cell suspensions were centrifuged at 200× *g* for 4 min and resuspended in complete NSC proliferation medium containing NeuroCult NSC basal medium and 10% NeuroCult NSC proliferation supplements (#05701, STEMCELL Technologies) with 20 ng/mL epidermal growth factor (EGF, AF-100-15, PeproTech, Rocky Hill, NJ, USA). Cells were cultured at 37 °C under humidified and 5% CO_2_ conditions. After primary neurospheres reached a diameter of 150–200 μm (at 4 days), cultures were dissociated with Accumax (Cat# AM105, Innovative Cell Technologies, San Diego, CA, USA) and replated at 150 cells/mm^2^. Genomic DNA was prepared from WT and *Mkp-1* KO NSCs using NucleoSpin^®^ Tissue kit (740952, MACHEREY-NAGEL, Düren, Germany) and genotyped as mentioned above to confirm *Mkp-1* knockout status (Figure S1). For differentiation of NSC, dishes, plates, or coverslips were coated with 200 μg/mL Poly-D-lysine (PDL, Sigma-Aldrich, St. Louis, MO, USA) at 4 °C overnight followed by 10 ng/μL laminin (L2020, Sigma-Aldrich) at room temperature for 2 h. After dissociated with Accumax, NSCs were plated at 400 cells/mm^2^ and cultured in complete differentiation medium containing 10% NeuroCult NSC differentiation supplements (#05703, STEMCELL Technologies) for future uses. For the treatment of cells, the autophagy activator, rapamycin (10 nM, dissolved in DMSO, 37094, Sigma-Aldrich), or the autophagy inhibitor, 3-methyladenine (3-MA, 2 mM, dissolved in medium, M9281, Sigma-Aldrich) was added after differentiation, and media was refreshed 24 h later. 

### 2.4. Quantitative PCR

RNA from undifferentiated and differentiated NSCs at different time points were prepared with E.Z.N.A.^®^ Total RNA Kit (SKU: R6834-01, Omega Bio-Tek, Norcross, GA, USA) and treated with DNase I (M0303S, New England Biolabs, Ipswich, MA, USA) followed by clean up with Monarch^®^ RNA Cleanup Kit (T2030S, New England Biolabs). cDNA was synthesized using the iScript cDNA Synthesis kit (1708890, Bio-Rad, Hercules, CA, USA). Quantitative PCR (qPCR) was performed with primers and exon spanning Taqman probes for mouse *Mkp-1* and β-actin with FAM-MGB and VIC-MGB, respectively (Mm00457274_g1/4331182 and Mm02619580_g1/ 4448489, ThermoFisher Scientific). qPCR was performed on a StepOnePlus Real-Time PCR System (ThermoFisher Scientific), and analyses were performed using β-actin as an internal reference gene for normalization in the ∆∆CT analyses. 

### 2.5. Immunocytochemistry

Cells cultured on coated coverslips were washed with PBS and fixed with 4% PFA at room temperature for 15 min. Then cultures were washed three times in PBS with 0.05% Triton X-100, followed by blocking at room temperature for 30 min in TBS-Blotto (0.15 M NaCl, 20 mM Tris-HCl, pH 7.5, 4.5% non-fat dry milk) with 0.1% Triton X-100. Cells were incubated with anti Tuj1 (1:1000, T8578, Sigma-Aldrich) and GFAP antibody (1:1000, #PA1-10019, ThermoFisher Scientific) for 1 h at room temperature with shaking, washed three times, and incubated with corresponding Alexa Fluor secondary antibodies (A11029, A11012, ThermoFisher Scientific) covered with foil for 1 h. Cells were rinsed twice, stained with 300 nM DAPI for 5 min, and rinsed twice. Coverslips were mounted with mowiol and imaged using the Lionheart FX automated microscope. Coverslips were coded, and the experimentalist was blind to their assignments. In total, 15–25 fields from 3–5 coverslips were scanned under 20× objective and counted using ImageJ [29]. 

### 2.6. Immunoblotting

Whole-cell lysates were collected using RIPA buffer (ThermoFisher Scientific) containing protease/phosphatase inhibitors (#5872, Cell Signaling Technology, Danvers, MA, USA). Cytoplasmic fraction was isolated from cells using the NE-PER™ Nuclear and Cytoplasmic Extraction Reagents (78833, ThermoFisher Scientific). Protein concentrations were measured on a Spectramax M5e multimode plate reader (Molecular Devices, San Jose, CA, USA) using the DC Protein Assay (5000111, Bio-Rad). For Western blotting, proteins were boiled at 95 °C for 5 min and loaded for sodium dodecyl sulfate polyacrylamide gel electrophoresis (SDS-PAGE). Blots were transferred onto polyvinylidene difluoride (PVDF) membranes (#1620177, Bio-Rad) followed by blocking with 5% non-fat milk. Membranes were incubated with antibodies against LC3 (1:500, APG8B, Abgent), BNIP3 (AP1312a, 1:500, Cell Signaling Technology), β-actin (AA5316, 1:5000, Sigma-Aldrich), or HSP90 (1:2500, C45G5, Cell Signaling Technology) at 4 °C overnight and HRP conjugated secondary antibodies (AC2115, AC2114, Azure Biosystems, Dublin, CA) for 2 h at room temperature. Membranes were briefly incubated in Clarity Western ECL substrate (#1705062, Bio-Rad) before chemiluminescent imaging with Azure c600 Gel Imaging System (Azure Biosystems). Densitometric analyses were performed using ImageJ gel analysis functions. Samples were normalized to β-actin (whole-cell lysis) or HSP90 (cytoplasmic fraction). 

### 2.7. Flow Cytometry

NSCs were seeded in 6-well plates in 3 mL of complete proliferation or differentiation medium. Prior to flow cytometry, cells were dissociated with Accumax and centrifuged for 5 min at 400× *g* at room temperature. Cells were then stained with autophagy/cytotoxicity Dual Staining Kit (600140, Cayman Chemical, Ann Arbor, MI, USA). In brief, monodansylcadaverine (MDC, a fluorescence marker of multilamellar autophagic vacuoles [30]) and propidium iodide (PI) were diluted at 1:1000 in Cell-Based Assay Buffer (came with the kit) to make the staining solution. For each sample, 350 μL staining solution was added and incubated at 37 °C for 10 min in the dark. Cells were centrifuged and washed with 500 μL Cell-Based Assay Buffer. Data of forward scatter (FSC), side scatter (SSC), Blue D (PI), and Violet B (MDC) channels were collected on a 3-laser, 12-color BD LSR-II platform (BD Biosciences, San Jose, CA, USA). Data were analyzed with FlowJo software (FLOWJO, Portland, OR, USA). Ten thousand events were collected per sample. Debris, clumped cells, and dead cells were eliminated by gating with FSC, SSC, and PI. The median values for MDC used as an indicator of autophagy were analyzed in single live cells and compared among different groups. 

### 2.8. Luciferase Assays

WT or *Mkp-1* KO NSCs were dissociated with Accumax and counted. Mouse neural stem cell NucleofectorTM kit (VPG-1004, Lonza AG, Cologne, Germany) was used for electroporation. Briefly, 2 × 10^6^ cells were collected and resuspended in 100 μL Mouse Neural Stem Cell Nucleofector^®^ Solution with supplement 1 and mixed with 2.5 μg pGL3-Basic based luciferase reporter vector and 0.25 μg pRL-TK control vector. The cell/plasmid suspension was transferred into a cuvette and inserted into the Nucleofector^®^ Cuvette Holder. Program A-033 was applied on a NucleofectorTM II (Amaxa Biosystems, Cologne, Germany). Then, 500 μL pre-equilibrated culture medium was added to the cuvette immediately. Cells were gently transferred into a prepared 24-well plate with complete differentiation medium. Twenty-four hours after transfection, cells were lysed, and luciferase activity measurements were performed with Dual-Luciferase^®^ Reporter Assay System (E1910, Promega, Madison, WI, USA) on a SpectraMax iD3 Microplate Reader (Molecular Device, San Jose, CA, USA). pGL3-Basic and pRL-TK were purchased from Promega. pGL3-Basic based Bnip3 promoter reporter vector (pGL3-Bnip3) was provided by Dr. Richard Bruick (UT Southwestern) [31].

### 2.9. siRNA Transfection

NSCs were dissociated with Accumax, and 5 × 10^6^ single cells were centrifuged and resuspended in 100 μL Mouse Neural Stem Cell Nucleofector^®^ Solution with supplement 1 and mixed with 50 pmol mouse Bnip3 siRNA or non-targeting siRNA (M-040256-01, D-001206-14-05, Dharmacon, Lafayette, CO, USA). Electroporated cells were allowed to recover in T25 flasks for 72 h prior to differentiation for downstream analyses.

### 2.10. Statistical Analysis

**Figure 1:**
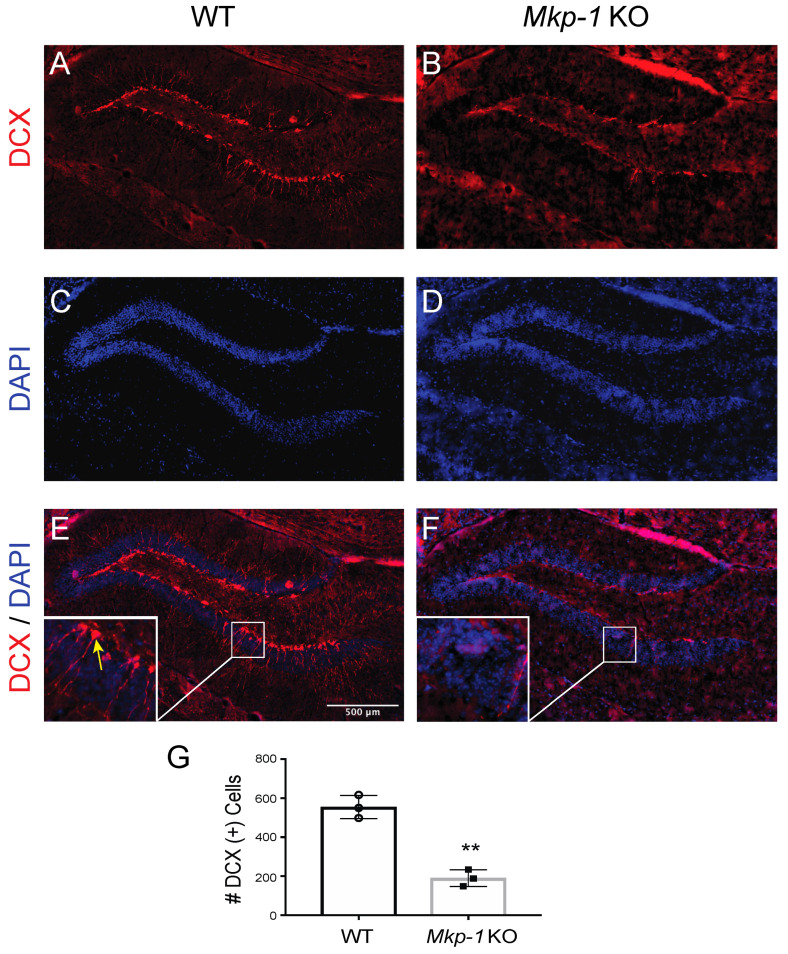
*Mkp-1* KO mice exhibit fewer DCX positive neurons in the dentate gyrus (DG). (**A**,**B**) Immunofluorescence images of the dentate gyrus in wild-type (WT) and *Mkp-1* KO mice stained with doublecortin (DCX). (**C**,**D**) DAPI nuclear counterstain of the dentate gyrus. (**E**,**F**) Composite images with high-power view illustrating individual DCX+ cells (yellow arrow). (**G**) Results of DCX+ cell counts in WT and *Mkp-1* KO cultures (*n* = 3, *t*-test, ** *p* < 0.01).

**Figure 2:**
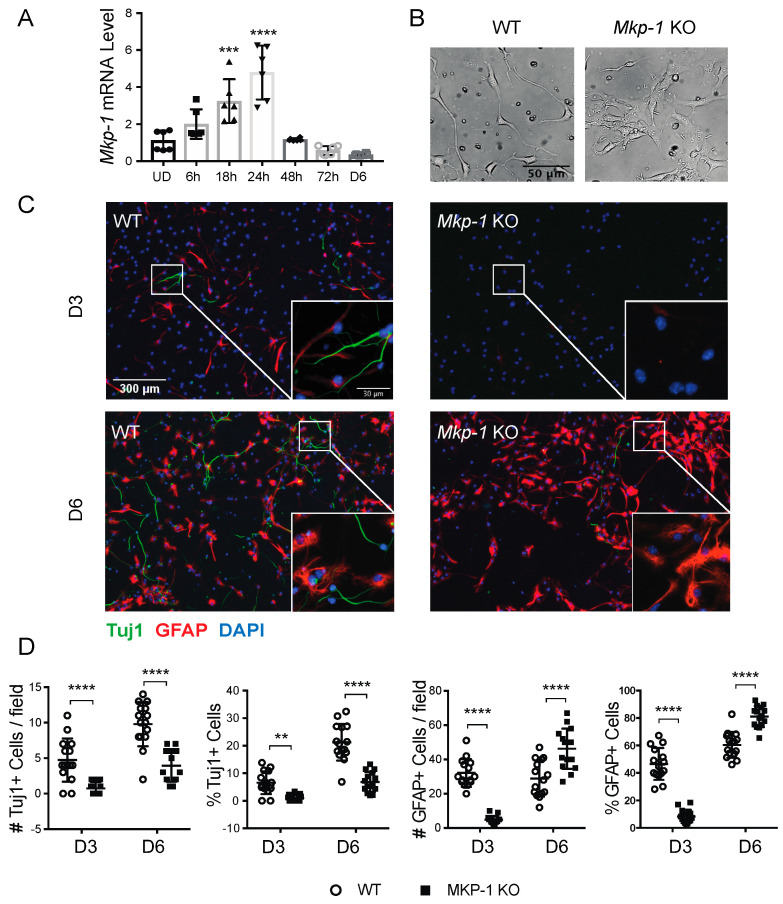
Loss of MKP-1 inhibits neurogenesis in differentiated NSC cultures. (**A**) Time course analysis of *Mkp-1* mRNA fold-change in expression in differentiated wild-type neural stem cells compared with undifferentiated (UD) cultures (*n* = 6, one-way ANOVA, *** *p* < 0.001, **** *p* < 0.0001). (**B**) Live-cell images of WT and *Mkp-1* KO neural stem cultures 24 h post-differentiation. (**C**) Immunocytochemical staining of differentiated WT and *Mkp-1* KO NSCs for Tuj1 (green) and GFAP (red) on day 3 (D3) and day 6 (D6) post-differentiation. (**D**) Absolute counts (#) per field and fraction (%) of Tuj1+ and GFAP+ cells relative to total cell # per field at D3 and D6 (*n* = 15, two-way ANOVA, ** *p* < 0.01, **** *p* < 0.0001).

**Figure 3:**
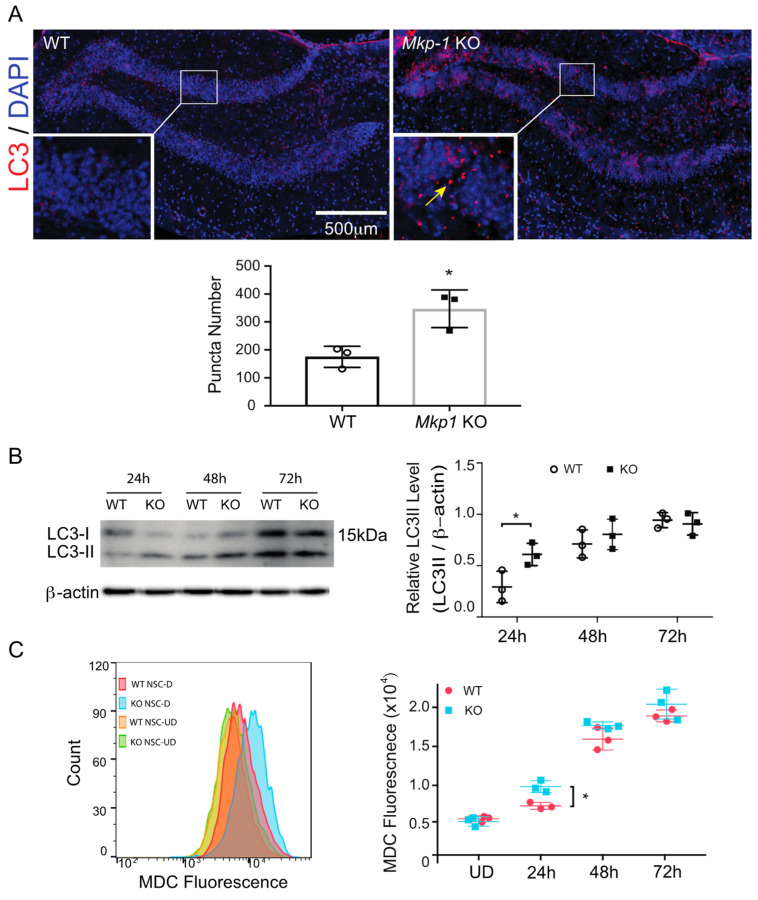
Loss of MKP-1 induces autophagy in the CNS and neural stem cultures. (**A**) LC3 immunofluorescence with DAPI counterstaining within the dentate gyrus of WT and *Mkp-1* KO mice. The inset illustrates the increased accumulation of LC3 puncta (yellow arrow) in *Mkp-1* KO mice. The number of puncta was compared between WT and *Mkp-1* samples (*n* = 3, *t*-test, * *p* < 0.05). (**B**) Western blotting (**Left**) and quantitative densitometry (**Right**) for LC3-II levels in neural stem cultures 24, 48, and 72 h post-differentiation in both genotypes (*n* = 3, two-way ANOVA, * *p* < 0.05). (**C**) Flow cytometry analyses of autophagy in NSCs stained with monodansylcadaverine (MDC). Representative MDC fluorescence plots from undifferentiated (UD) and differentiated (D) NSCs (**Left**) and results comparing the effect of genotype on the median of MDC fluorescence over time (**Right**) are shown (*n* = 3, two-way ANOVA, * *p* < 0.05).

**Figure 4:**
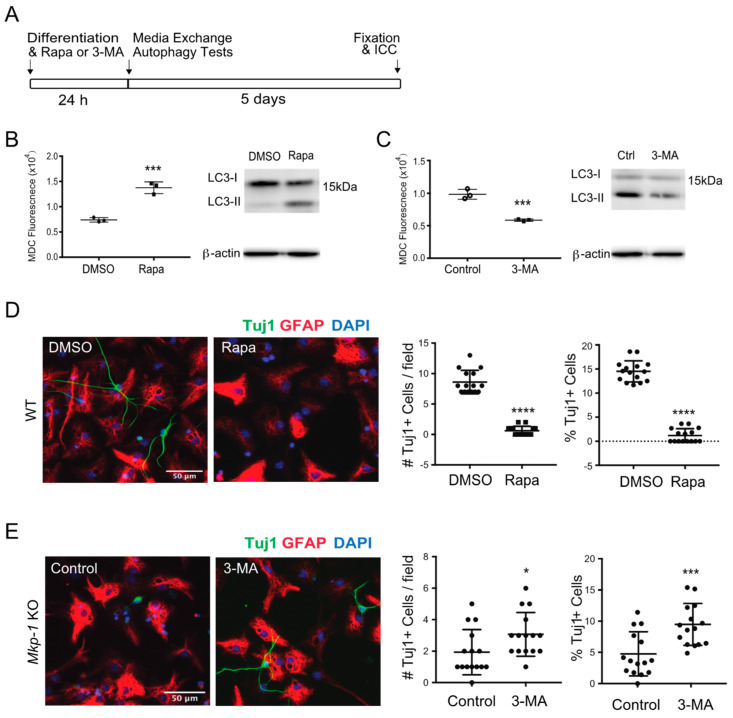
Pharmacological manipulation of autophagy influences neurogenesis. (**A**) Experimental timeline illustrating the sequence of NSC differentiation and drug treatment, media exchange, and ICC endpoint analysis. (**B**) Autophagy level of WT NSC-D 24 h post differentiation with DMSO or rapamycin in DMSO (Rapa, 10 nM) using average MDC fluorescence by flow (left) and LC3II immunoblotting. (**C**) Changes of MDC fluorescence and LC3II level in *Mkp-1* KO NSC-D 24 h after differentiation with or without the treatment of 3-MA (2 mM, dissolved in medium). (**D**) Effect of DMSO vs. rapamycin on the number (#) per field and fraction (%) of Tuj1+ neurons (green) relative to total cell # on day 6 in WT NSC-D cultures. (**E**) Effect of 3-MA treatment on the number (#) per field and fraction (%) of Tuj1+ neurons (green) relative to total cell # on day 6 of *Mkp-1* KO NCS-D cultures (*n* = 15, *t*-test, * *p* < 0.05, *** *p* < 0.001, **** *p* < 0.0001).

**Figure 5:**
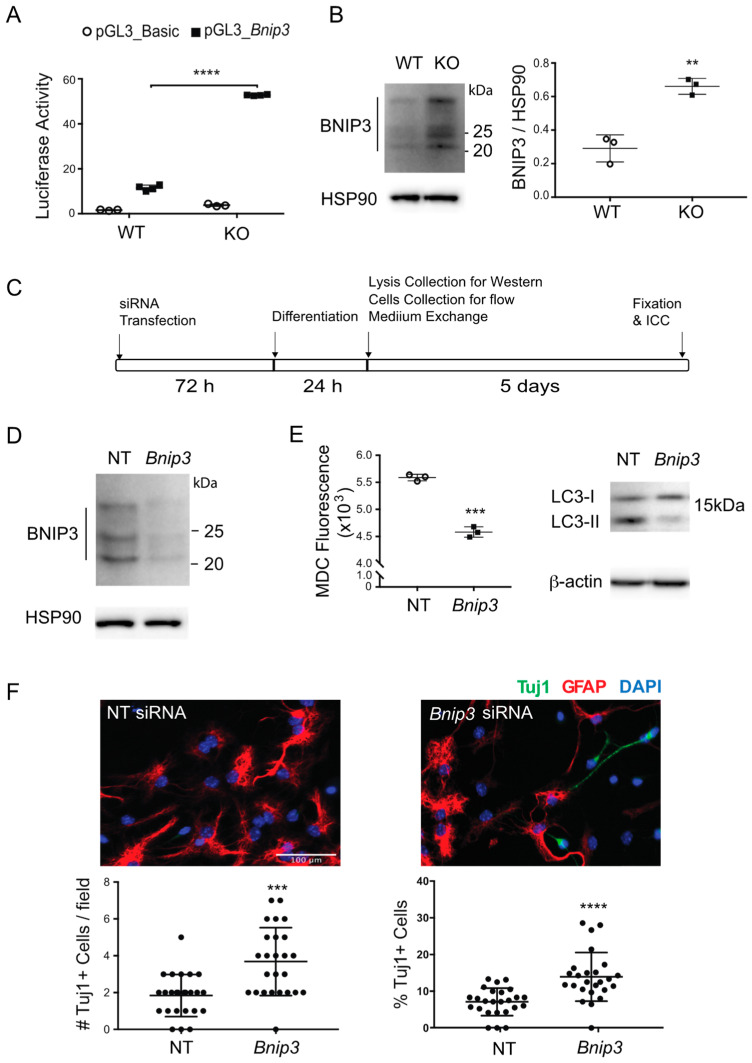
BNIP3 induces autophagy and represses neurogenesis in *Mkp-1* KO NSCs. (**A**) Relative activity of pGL3_Bnip3 vs. control pGL3_Basic reporter constructs in differentiated (24 h) WT and *Mkp-1* KO NSCs (*n* = 4, two-way ANOVA, ****p < 0.0001). (**B**) Protein blotting for BNIP3 expression in the cytoplasmic fraction of NSCs 24 h post-differentiation (Left) and the quantification by densitometry relative to the loading control HSP90 (right) (*n* = 3, *t*-test, ** *p* < 0.01). (**C**) Experimental timeline for Bnip3 knockdown illustrating sequential siRNA treatment, differentiation, and various endpoint analyses. (**D**) Confirmation of BNIP3 knockdown in *Mkp-1* KO cells 24 h post-differentiation by protein blotting (NT, non-targeting siRNA transfection; Bnip3, mouse *Bnip3* siRNA transfection). (**E**) Changes of MDC and LC3II levels in *Mkp-1* KO NSC-D with *Bnip3* siRNA transfection using flow cytometry and immunoblotting, respectively, 24 h after differentiation (*n* = 3, *t*-test, *** *p* < 0.001). (**F**) Absolute number (#) per field and fraction (%) of Tuj1+ neurons (green) relative to total cell # following Bnip3 siRNA transfection in *Mkp-1* KO NSC-D on day 6 post differentiation (*n* = 25, *t*-test, *** *p* < 0.001, **** *p* < 0.0001).

Statistical analyses were performed using GraphPad Prism^®^v7 (GraphPad Software, San Diego, CA, USA). Data were expressed as mean ± standard deviation and compared among groups, accepting *p* < 0.05 as significant. Unpaired two-tailed *t*-testing was used to analyze data from Figures 1, 3A, 4 and 5B,E,F. Data from Figures 2D, 3B,C and 5A were analyzed using two-way ANOVA with Sidak’s multiple comparison post hoc test. The qPCR data shown in Figure 2A was analyzed by one-way ANOVA with Tukey’s multiple comparison post hoc test.

## 3. Results

### 3.1. Loss of MKP-1 Has a Suppressive Effect on Neurogenesis 

The balance between MAPK and MKP activity regulates synaptic plasticity and related developmental processes within the central nervous system [32,33,34]. To evaluate the potential influence that MKP-1 has on neurogenesis, we first assessed whether loss of MKP-1 function altered the number of doublecortin (DCX) positive neurons within the dentate gyrus (DG). Results indicated that DCX counts were reduced in *Mkp-1* KO mice relative to WT littermates (WT 555 ± 60 vs. KO 190 ± 43, *n* = 3, *p* < 0.01) (Figure 1).

We next investigated the effect of differentiation on endogenous *Mkp-1* mRNA expression using an in vitro model of neural stem cell differentiation cultures (NSC-D). Differentiation induced *Mkp-1* mRNA expression in WT NSCs, which peaked at 24 h and returned to baseline after 48 h (Figure 2A). Differentiated NSCs were also morphologically distinct, with WT NSCs producing process-bearing cells, while *Mkp-1* KO NSCs grew in clusters with less distinct morphologies (Figure 2B). ICC analyses revealed that by 3 days post-differentiation, WT NSCs contained both class III beta-tubulin (Tuj1) positive neurons or glial fibrillary acidic protein (GFAP) positive cells, representing either radial glia or astroglia. In contrast, *Mkp-1* KO cultures produced fewer Tuj1 or GFAP-positive cells by day 3 (Figure 2C,D). While the expression of both markers increased by day 6, the number and percentage of Tuj1+ cells in *Mkp-1* KO NSC-D cultures were reduced compared with WT (number/field, WT 9.8 ± 3.1 vs. KO 3.9 ± 2.3, *n* = 15, *p* < 0.0001; percentage, WT 21.3 ± 6.6% vs. KO 6.8 ± 3.6%, *n* = 15, *p* < 0.0001), which was offset by an increase in the number/field (WT 28.9 ± 10.6 vs. KO 46.3 ± 11.7, *n* = 15, *p* < 0.0001) and percentage (WT 60.5 ± 9.4% vs. KO 81.1 ± 7.7%; *n* = 15, *p* < 0.0001) of GFAP+ cells. These data indicated that MKP-1 expression regulates neurogenesis in differentiated neural stem cultures. 

### 3.2. Loss of MKP-1 Induces Autophagy in Differentiated NSCs

Autophagy influences both the proliferation and differentiation of stem cell populations [35]. There is also ample evidence to indicate that autophagic responses exhibit cell-specific differences [17,18]. Given these observations, we next evaluated MKP-1 effects on autophagy in the CNS, tracking the abundance of intracellular LC3 puncta considered an established marker of autophagy [36]. *Mkp-1* KO mice exhibited a robust increase in the number of LC3 puncta present in the dentate gyrus compared to WT controls (WT, 175 ± 38 vs. *Mkp-1* KO 346 ± 67, *n* = 3, *p* < 0.05) (Figure 3A). Similarly, LC3-II expression was increased in *Mkp-1* KO cultures compared to WT NSCs 24 h post-differentiation. This difference declined between 48 and 72 h (Figure 3B), mirroring changes in *Mkp-1* expression. We observed similar trends in cultures labeled with monodansylcadaverine (MDC), a fluorescence marker of multilamellar autophagic vacuoles. While differentiation induced MDC levels in both genotypes, MDC levels were higher in *Mkp-1* KO cultures relative to WT controls (KO 9841 ± 765 vs. WT 7382 ± 437, *n* = 3, *p* < 0.05) (Figure 3C) 24 h post-differentiation. This effect normalized within 48 h, consistent with observed changes in LC3-II levels. These results indicate that MKP-1 influences levels of autophagy early in the process of NSC differentiation.

### 3.3. Effect of MKP-1 Deficiency on Neurogenesis Is Autophagy-Dependent

Given the observed effects of MKP-1 on neurogenesis and autophagy, we next asked whether MKP-1’s effects on neurogenesis were autophagy-dependent. To do this, we treated WT NSC-D cultures with the autophagy-inducer rapamycin (10 nM, in DMSO), while exposing *Mkp-1* KO cultures to the autophagy inhibitor 3-MA (2 mM, in medium) during the first 24 h of differentiation (Figure 4A). Drug effects on levels of autophagy were confirmed in NSC-D cultures using MDC fluorescence and LC3-II expression (Figure 4B). When WT NSC-D cultures were exposed to rapamycin, both MDC and LC3-II levels were increased relative to DMSO treated controls. Conversely, treatment of *Mkp-1* KO cultures with 3-MA treatment decreased both MDC and LC3-II levels when compared against controls (Figure 4C). 

To evaluate drug effects on day 6 NSC-D cultures, we performed ICC staining for the neuronal marker Tuj1. Results indicated that compared with DMSO controls, rapamycin reduced the number/field (DMSO 8.6 ± 1.9 vs. Rapa 0.6 ± 0.7, *n* = 15, *p* < 0.0001) and percentage (DMSO 14.5 ± 2.2% vs. Rapa 1.2 ± 1.4%, *n* = 15, *p* < 0.0001) of Tuj1+ neurons in WT NSC-D cultures (Figure 4D). Conversely, compared with controls, 3-MA increased the number/field (control 1.9 ± 1.4 vs. 3-MA 3.1 ± 1.4, *n* = 15, *p* < 0.05) and percentage (control 4.7 ± 3.5% vs. 3-MA 9.3 ± 3.3%, *n* = 15, *p* < 0.001) of Tuj1+ neurons in *Mkp-1* KO cultures (Figure 4E). These findings indicate that MKP-1-dependent regulation of autophagy during the early stages of NSC differentiation exerts important effects on neurogenesis.

### 3.4. BNIP3 Regulates Autophagy and Neurogenesis in Mkp-1 KO NSCs

Initially described as a pro-death, hypoxia-inducible target, the mitochondrial protein BCL2 interacting protein 3 (BNIP3) promotes autophagy under stress conditions [33,34]. To investigate whether the loss of MKP-1 was associated with changes in BNIP3 expression, we conducted luciferase reporter assays using the vector pGL3-Bnip3 containing the proximal Bnip3 promoter [31]. By 24 h post-differentiation, pGL3-Bnip3-transfected *Mkp-1* KO NSC-D cultures exhibited increased reporter activity relative to WT cultures (WT 11.5 ± 1.3 vs. KO 52.8 ± 0.3, *n* = 4, *p* < 0.0001) (Figure 5A). Increased expression of BNIP3 protein in lysates generated from *Mkp-1* KO NSC-D cultures was confirmed by Western blotting (Figure 5B). Furthermore, siRNA knockdown of BNIP3 was associated with reductions in both MDC signal (NT 5588 ± 61 vs. Bnip3 4582 ± 96, *n* = 3, *p* < 0.001) and levels of LC3-II in *Mkp-1* KO cultures (Figure 5C–E). Western blotting confirmed the efficiency of siRNA-mediated effects on BNIP3 suppression relative to non-targeting siRNA (NT) treated controls (Figure 5D). 

While BNIP3-dependent effects on autophagy and apoptosis in neurons have been described [37], their role in neurogenesis remains untested. We found that siRNA mediated Bnip3 knockdown enhanced the generation of Tuj1+ cells in *Mkp-1* KO NSC-D cultures (number/field, NT 1.8 ± 1.1 vs. Bnip3 3.7 ± 1.8, *n* = 25, *p* < 0.001; percentage, NT 7.1 ± 3.8% vs. Bnip3 13.9 ± 6.6%; *n* = 25, *p* < 0.0001) (Figure 5F). These results indicate downregulation of BNIP3 abrogated *Mkp-1* KO’s observed effects on neurogenesis. Collectively, these results suggest that MKP-1’s effects on autophagy are BNIP3-dependent. Additionally, besides enhancing autophagy in NSC-D cultures, BNIP3 induction in *Mkp-1* KO NPCs exerts a repressive effect on neurogenesis. 

## 4. Discussion

These studies indicate that the canonical nuclear MAPK phosphatase MKP-1 exerts pro-neurogenic bias via effects on BNIP3 expression and autophagic signaling. We found that MKP-1 loss of function is associated with impaired neurogenesis in the dentate gyrus and differentiated NSC cultures under basal conditions. The reduced capacity for neurogenesis in differentiated *Mkp-1* KO cultures correlates with increased autophagy and BNIP3 expression. Conversely, *Bnip3* knockdown in *Mkp-1* KO cultures rescues autophagy and neurogenic activity. 

Mounting evidence indicates that MKP-1 serves a protective role in the CNS under a range of disease-relevant conditions. MKP-1 promotes the growth and elaboration of neuronal processes in dopaminergic neurons cultured from E14 rat ventral mesencephalon, protecting them against neurotoxic challenge [38]. MKP-1 also exerts neuroprotective effects following acute cerebral ischemia and in chronic models of neurodegenerative disease [33,39]. For example, MKP-1 loss of function accelerates pathogenic changes in models of Alzheimer’s disease, characterized by impaired neurogenesis. Conversely, MKP-1 induction improves long-term potentiation and corrects cognitive deficits in this disease [20]. In light of our observations regarding the pro-neurogenic potential of MKP-1, it is plausible that stress-induced activation of MKP-1 reflects a compensatory mechanism intended to promote adaptive plasticity within hippocampal networks [40]. 

The existing literature supports a role for MKP-1 in regulating autophagy in non-neuronal contexts. In 2016, Wang et al. [17] reported *Mkp-1* shRNA knockdown increased basal and rapamycin-increased autophagic flux in murine embryonic fibroblasts and the human CAOV3 ovarian line. Our finding that loss of *Mkp-1* increased autophagy in the dentate gyrus and in differentiated NSCs is consistent with these reports. An important aspect of this study is that MKP-1’s effect on autophagy appears operative during the initial 24 h post-differentiation, consistent with transient *Mkp-1* mRNA expression in differentiated wild-type NSC cultures. These findings suggest that early induction of MKP-1 and repression of autophagy may be required to exert the observed pro-neurogenic effect. In other studies, transplanted GABAergic interneuron precursors exhibit marked sensitivity to autophagy within the first 24 h following transplantation. Inhibition of autophagy increases the survival and fraction of neurite-bearing GABAergic interneurons within two weeks post-implantation [41]. While our in vitro analyses focused on the initial post-differentiation phase (3–6 days), our in vivo analyses suggest that the pro-neurogenic effects of basal MKP-1 activity in the dentate gyrus persist into adulthood. By extension, these findings argue that targeted augmentation of MKP-1 activity could be used to treat neurodegenerative conditions or acute brain injury by promoting the neurogenic potential of endogenous or transplanted stem progenitors [42,43]. 

Initially described by Daido et al. [44], BNIP3 -dependent control over autophagy has been demonstrated in a range of cell types, including neurons and myocytes [37,45]. Clues from the existing literature suggested several options through which MKP-1 might influence BNIP3 activity. For example, MKP-1 is associated with changes in JNK-dependent phosphorylation of BNIP3 [19]. However, while we found that λ-phosphatase treatment induced a minor shift in the high molecular weight BNIP3 species in NSC-D cultures (Supplementary Figure S2A), no genotype-specific differences were seen in these two forms under basal conditions. Likewise, loss of MKP-1 in NSCs had no apparent effect on levels of JNK or p38 activation within 24 h of differentiation (Supplementary Figure S2B,C). The lack of MKP-1-dependent effects on these markers may relate to cell-context dependent effects and functional redundancy between the MAPK and DUSP family members [18]. MKP-1 also inhibits the accumulation of HIF-1α protein [46,47], and HIF-1α induced expression of BNIP3 in the context of ischemia-reperfusion injury induces autophagy [48]. Although basal expression of HIF-1α protein remained low in NSC-D cultures irrespective of *Mkp-1* status (Supplementary Figure S2D), BNIP3 expression was increased in *Mkp-1* KO cultures (Figure 5A,B). Of note, it remains possible that *Mkp-1* deletion increased HIF-1α’s transcriptional potency without influencing its stability, which is typically low under normoxic conditions. Nonetheless, we suspect that BNIP3 induction contributed to enhanced autophagy by either interaction with different molecules such as LC-3 and its related molecular receptors [49] or by forcing the release of Beclin-1 from heterodimeric complexes with BCL-2 [50], suppression of the GTPase Rheb, and inhibition of mTOR [51]. 

Our findings link MKP-1 deficiency with BNIP3-induced autophagy and repressed neurogenesis in differentiated NSCs. Given BNIP3’s established role in autophagic cell death [44], it is tempting to posit that BNIP3’s anti-neurogenic effects are autophagy-related, acting via pathways controlling autophagy and apoptosis, which exhibit significant crosstalk. For example, the ATG5-ATG12 conjugation system is required for the process of autophagosome formation, while ATG5 and ATG12 regulate stress-induced apoptosis [52]. It is also plausible that alternate BNIP3 targets may be involved. For example, the pro-survival factors BCL-2 and BCL-XL regulate neural differentiation, support the survival of newborn neurons, and increase rates of adult neurogenesis [53,54,55,56,57]. Considering that BNIP3 induces cell-death by disrupting BCL-2 and BCL-XL interactions [58], elevated BNIP3 levels could titrate out these pro-survival BH3 domain proteins with repressive effects on neurogenesis.

Since our in vitro endpoint analyses focused on changes occurring during the first week, it remains to be seen whether MKP-1 exerts delayed effects on NSC differentiation. For example, manipulation of MKP-1 could influence the engraftment and differentiation of transplanted NSC populations. It is also important to consider that neurogenic effects observed in the *Mkp-1* KO background may reflect contributions from non-cell-autonomous effects (i.e., paracrine or cell–cell interactions). For example, since the loss of MKP-1 enhances MAPK-dependent inflammation [59,60], the secretion of anti-neurogenic factors like FGF2 or IL-1β from non-neuronal CNS cell types could suppress neurogenesis in vivo. Implementation of a conditional *Mkp-1* knockout model would provide insight regarding this nuanced aspect of MKP-1 function in the CNS [61]. 

## 5. Conclusions

We report the essential role of the dual-specificity phosphatase MKP-1 in neurogenesis during a critical window of neural stem cell (NSC) differentiation. MKP-1 induction within 24 h after NSC differentiation exerts a pro-neurogenic bias via effects on BNIP3 and autophagy. Specifically, MKP-1 deficiency enhances BNIP3 expression and autophagy and impairs neurogenesis in vivo and in differentiated NSCs. Conversely, selective knockdown of BNIP3 is sufficient to reverse these changes. Collectively, these findings argue that transient manipulation of intrinsic MKP-1 activity during the initial phase of NSC differentiation could be used to improve the therapeutic potential of stem cell-based protocols. 

## Data Availability

Data for this study can be found in the manuscript.

## Supplementary Materials

The following are available online at https://www.mdpi.com/article/10.3390/biom11121871/s1, Figure S1: Genotyping of mice and neural stem cells, Figure S2: Analyses of loss of MKP-1 function on BNIP3, JNK, and p38 phosphorylation and HIF-1α expression in differentiated neural stem cultures.
