# Supplementary figures and images for: The MAP Kinase Phosphatase MKP-1 Modulates Neurogenesis via Effects on BNIP3 and Autophagy
Source: Biomolecules. 2021 Dec 14;11(12):1871. doi: 10.3390/biom11121871 (PMC8699509; doi:10.3390/biom11121871)

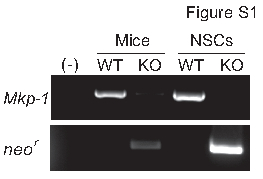

Supplement: Supplementary file 1 [file biomolecules-11-01871-s001.zip › Figure S1.jpg]

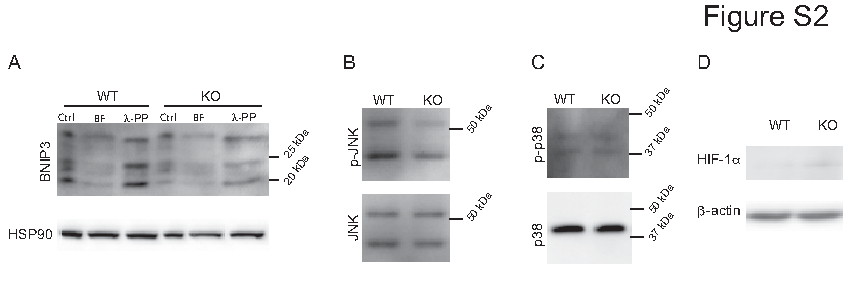

Supplement: Supplementary file 1 [file biomolecules-11-01871-s001.zip › Figure S2.jpg]
